# Systems crosstalk between antiviral response and cancerous pathways via extracellular vesicles in HIV-1-associated colorectal cancer

**DOI:** 10.1016/j.csbj.2023.06.010

**Published:** 2023-06-12

**Authors:** Zimei Chen, Ke Yang, Jiayi Zhang, Shufan Ren, Hui Chen, Jiahui Guo, Yizhi Cui, Tong Wang, Min Wang

**Affiliations:** a The First Affiliated Hospital, MOE Key Laboratory of Tumor Molecular Biology, Institute of Life and Health Engineering, College of Life Science and Technology, Jinan University, Guangzhou, Guangdong 510632, China; b Department of Infectious Diseases, Institute of HIV/AIDS, The First Hospital of Changsha, Changsha, Hunan 410005, China

**Keywords:** HIV-1-associated colorectal cancer, Proteomics, HIV-1 reservoir, Extracellular vesicles, IFN pathway, Cell-associated HIV-1 RNA, Antiviral response

## Abstract

HIV-1 associated colorectal cancer (HA-CRC) is one of the most understudied non-AIDS-defining cancers. In this study, we analyzed the proteome of HA-CRC and the paired remote tissues (HA-RT) through data-independent acquisition mass spectrometry (MS). The quantified proteins could differentiate the HA-CRC and HA-RT groups per PCA or cluster analyses. As a background comparison, we reanalyzed the MS data of non-HIV-1 infected CRC (non-HA-CRC) published by CPTAC. According to the GSEA results, we found that HA-CRC and non-HA-CRC shared similarly over-represented KEGG pathways. Hallmark analysis suggested that terms of antiviral response were only significantly enriched in HA-CRC. The network and molecular system analysis centered the crosstalk of IFN-associated antiviral response and cancerous pathways, which was favored by significant up-regulation of ISGylated proteins as detected in the HA-CRC tissues. We further proved that defective HIV-1 reservoir cells as represented by the 8E5 cells could activate the IFN pathway in human macrophages via horizonal transfer of cell-associated HIV-1 RNA (CA-HIV RNA) carried by extracellular vesicles (EVs). In conclusion, HIV-1 reservoir cells secreted and CA-HIV RNA-containing EVs can induce IFN pathway activation in macrophages that contributes to one of the mechanistic explanations of the systems crosstalk between antiviral response and cancerous pathways in HA-CRC.

## Graphical Abstract

**Figure:**
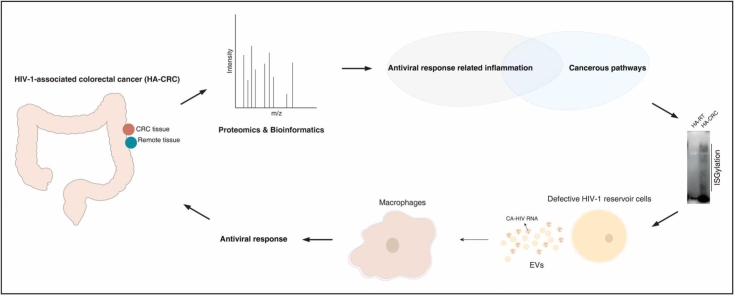

## 1 Introduction

According to the World Health Organization, there were approximately 38.4 million people living with HIV (PLWH) globally in 2021. Since the first HIV-1-infected case was reported in 1981, numerous cancer types had been found to predominantly occur in PLWH, including Kaposi’s sarcoma, primary central nervous system lymphoma, non-Hodgkin lymphoma, and invasive cervical cancer [1], [2]. These cancer types were classified as AIDS-defining cancers (ADC). Since the highly active antiretroviral therapy (HAART) was introduced, ADC incidence had swiftly declined; however, non-AIDS-defining cancers (NADC) were becoming a major cause of morbidity and mortality [3], [4]. For example, Yuan et al. found that as compared with the general population, 30 out of the 40 NADC types, such as lung, brain and small intestine cancers, showed increased standardized incidence rates (SIR) in PLWH [5].

Multiple theories and hypotheses have been proposed to explain the mechanisms of developing NADC. In general, immunosuppression, oncogenic viral infection, chronic inflammation, pro-oncogenic effect of HIV, and drug influence were deemed potentially primary factors [6], [7], [8]. In this context, the -omics strategy has shown its significance. For example, regarding HIV-1 associated lung cancer (HALC), Wu et al. [9] and Zheng et al. [10] independently performed transcriptomic analyses on paired tumor/adjacent tissues from 5 and 17 subjects, respectively. They revealed over-represented pathways of mitosis and drug response in HALC. Bao et al. performed RNA-seq analysis on 7 paired tissues of HIV-1-infected kidney cancer, in which they highlighted vascular endothelial growth factor-activated receptor, IgG binding, and lipopolysaccharide receptor activities in this NADC [11].

HIV-1-associated colorectal cancer (HA-CRC) is one of the most understudied NADCs. It should be mentioned that differential conclusions had been made regarding the risk of CRC in PLWH. For example, in a British Columbia Hepatitis Testers Cohort study on 658,697 individuals, those with HIV-1 mono-infection were found to have significantly higher risk of CRC, with the hazard ratio (HR) of 2.30 [12]. Numerous other studies favored the increased risk of CRC in PLWH, including a meta-analysis on publications of 1985–1998 (SIR, 1.41) [13], a prospective study in US (SIR, 2.3) [14], and an Asian cohort follow-up study (SIR, 2.1) [15]. In contrast, there were reports showing that CRC had less or similar incidence in PLWH, including meta-analyses on publications of 1985–1999 (SIR, 0.45) [16], 1981–2004 (SIR, 0.81) [17] and 1992–2022 (SIR, 1.09) [5], as well as a US cohort study (1996–2012, SIRs, 0.51–0.69) [18]. But CRC screening was found to be significantly under-utilized in PLWH, serving as a possible factor for reaching the above-mentioned inconsistent conclusions on HA-CRC [19], [20].

Clinical Proteomics Tumor Assessment Consortium (CPTAC https://pdc.cancer.gov/pdc/study/PDC000116) published proteogenomics analysis on tissues from 96 subjects with CRC, in which the critical role of Rb phosphorylation and glycolysis pathways were revealed [21]. HA-CRC could be classified as a special CRC subtype. Therefore, in this study, we reported the first proteomic analysis on HA-CRC in comparison with the non-HA-CRC (CPTAC dataset) to explore their differential mechanisms in terms of systems biology and biological verifications.

## 2 Materials and methods

### 2.1 Participants and tissue sample acquisition

This study was approved by the ethics review committees of The First Hospital of Changsha and written informed consents were obtained from all participants. In total, seven patients were enrolled in this study. Tissue samples from Donors 1–4 were subjected to the proteomics study, while samples from Donors 5–7 were freshly obtained in the year of 2022 for biological verifications. All donors were diagnosed with primary and treatment-naïve HA-CRC, and their CRC staging was determined based on the 2021 version of Chinese Society of Clinical Oncology (CSCO) guidelines for CRC [22]. All donors had received HAART for 1–10 years upon enrollment, and their HIV-1 RNA viral load were all continuously suppressed to< 50 copies/mL. Detailed clinical characteristics of participants can be found in Table 1.

**Table 1:** Clinical characteristics of HA-CRC donors.

Donor	Gender	Age	Year (s) since HIV-1 infection	Histopathologic type	TNM^a^	Stage	Tumor site	Size^b^
1	Male	65	10	Moderately differentiated adenocarcinoma	T3N1cM0	IIIB	Sigmoid colon	3.4
2	Female	55	5	Moderately differentiated adenocarcinoma	T3N2aM0	IIIB	Transverse colon	5.0
3	Male	51	1	Moderately differentiated adenocarcinoma	T3N1cM0	IIIB	Rectum	2.5
4	Male	45	5	Poorly differentiated adenocarcinoma	T4bN1aM0	IIIC	Descending colon	4.5
5	Female	55	0.3	Moderately differentiated adenocarcinoma	T3N0M0	IIA	Sigmoid colon	9.0
6	Male	68	0.7	Moderately differentiated adenocarcinoma	T2N0M0	I	Rectum	1.7
7	Female	70	1	Moderately differentiated adenocarcinoma	T3N1aM0	IIIB	Rectum	1.0

a TNM staging was performed per the Chinese Society of Clinical Oncology (CSCO) guideline (version 2021) for colorectal cancer [22].

b Size indicates the tumor’s maximum diameter (cm).

HA-CRC tissues and HIV-1 associated remote tissues (HA-RT,>2-cm-away from the CRC tumor edge) were obtained during surgical resection. HA-CRC samples were subjected to postoperative pathological examination by professional pathologists. As we previously reported [23], the tumor cellularity, which was estimated as the ratio of tumor cells to all intertumoral cells, should be>85% for subsequent mass spectrometry (MS) analyses. After surgical resection, tissue samples were freshly frozen in liquid nitrogen, followed by storage at −80 ℃ prior to analyses.

### 2.2 Protein extraction

As we previously described (Luo et al., 2017; Zhang et al., 2017), tissues were washed for 3 times with 4 ℃ PBS (phosphate buffered saline, pH 7.4, ThermoFisher Scientific, Shanghai, China) and treated with ice-cold SDS lysis buffer (Beyotime, Nanjing, China) supplemented with 1 mM phenylmethanesulfonyl fluoride (PMSF, Sigma-Aldrich, Shanghai, China), protease inhibitor cocktail and phosphatase inhibitor (Roche, Shanghai, China). Tissues were then cut into small pieces and homogenized with a FastPrep-24 instrument (MP Biomedicals, Shanghai, China) for 30 s at the speed of 6.0 m/s; after an interval incubation at − 20 ℃ for 10 min, a repeated homogenization was performed. To disrupt DNA, supernatants were subjected to sonication (1 s on and 2 s off, 720 W, 10 min on ice) with a non-contact ultrasonic cell pulverizer (Scientz08-III, Ningbo, Zhejiang, China). To remove debris, the lysate was centrifuged for 30 min at 15,500g, 4 °C and the supernatant was collected in a new tube. Supernatant protein concentrations were determined by a BCA protein assay kit following the manufacturer’s instructions (Thermo).

### 2.3 Protein digestion

Protein digestion was performed as we previously described [24], [25], [26]. In brief, the extracted proteins were reduced (8 M Urea and 50 mM DTT at 37 °C, 1 h) and alkylated (100 mM IAA, at room temperature, 30 min) in 1.5 mL tubes. In-solution tryptic digestion was then performed by using the filter-aided sample preparation (FASP) method, exactly as the procedure in our previous reports [24], [25], [26].

### 2.4 High-pH RPLC fractionation

Peptides were rehydrated with deionized water containing 0.1% (v:v) formic acid and the peptide concentration was measured by the Pierce™ Quantitative Peptide Assays (Thermo). Peptides were then fractionated by a Waters XBridge® C18 5 µm column (4.6 × 250 mm) on LC-10 HPLC System (Wufeng, Shanghai, China) with mobile phases A (20 mM Ammonium formate, pH = 10) and phases B (phases A: acetonitrile = 1:5 (v:v)). Peptides were fractionated with a 44-min gradient elution at a speed of 0.8 mL/min with 5–30% phases B for 22 min, 30–42% phases B for 17 min and 42–65% phases B for 5 min. The eluate was collected every 2 min and 22 fractions were collected that were further pooled into 8 fractions. These pooled fractions were dried with a speed-vac, rehydrated with 300 μL 0.5% trifluoroacetic acid and desalted with Mono Tip™ C18 Pipette Tips (GL Sciences, Shanghai, China).

### 2.5 Mass spectrometry analysis

MS analysis was performed as we previously described with minor modifications [27], [28]. Peptides were analyzed in the data-dependent acquisition (DDA) to build spectral library, followed by the data-independent acquisition (DIA) MS analysis on individual samples. LC-MS/MS instrument used in this study was an Orbitrap Fusion Lumos Tribrid mass spectrometer equipped with an EASY-nanoLC 1200 HPLC system (Thermo). To allow retention time calibration, the iRT-standard (Biognosys, Schlieren, Switzerland) was added into the peptide solution at the volume ratio of 1:10. The DDA MS parameters were set as follows. (1) MS parameters: detector type, orbitrap; orbitrap resolution, 120000; scan range, 350–1350 *m/z*; RF lens, 40%; automatic gain control (AGC) target, 400000; maximum injection time, 50 ms. (2) MS/MS parameters: isolation mode, quadrupole; activation type, HCD; collision energy, 32%; detector type, orbitrap; orbitrap resolution, 15000; mass range, normal; AGC target, 50000; maximum injection time, 22 ms. The DIA MS parameters were set as follows. (1) MS parameters: detector type, orbitrap; orbitrap resolution, 120000; scan range, 350–1250 *m/z*; RF lens, 40%; AGC target, 400000; maximum injection time, 50 ms. (2) MS/MS parameters: isolation mode, quadrupole; activation type, HCD; collision energy, 32%; detector type, orbitrap; orbitrap resolution, 30000; mass range, normal; scan range: 200–2000 *m/z*; AGC target, 500000; maximum injection time, 50 ms.

### 2.6 Database searches

To generate DDA-based spectral library for DIA, we employed the Spectronaut Pulsar (version 14.10, Biognosys, Switzerland) to generate library by searching DDA raw files against a combined fasta database containing human (downloaded from Swiss-Prot on Aug 1, 2022, 20386 entries) and HIV-1 (downloaded from Swiss-Prot on Aug 1, 2022, 381 entries) proteins, as well as the iRT standard peptide sequences. Searching parameters included: enzyme, trypsin; max peptide length, 52; min peptide length, 9; missed cleavages, 2; fixed modifications, carbamidomethyl (C); variable modifications, acetyl (N-term), deamidation (NQ), oxidation (M); data filtering, Qvalue; precursor Qvalue cutoff, 0.01; protein Qvalue cutoff, 0.01; quantity MS-level, MS2; cross run normalization, true; normalization strategy, global normalization.

DIA raw files were then imported into Spectronaut to match the DDA-based spectral library. In the report section of Spectronaut, protein identification and quantitation information was exported for further analyses.

### 2.7 Gene set enrichment analysis (GSEA)

The GSEA software (version 4.2.3) was used to analyze the whole proteome against the hallmark gene sets (version 2022.1)[29], [30]. The R package clusterProfiler (version 4.4.4) [31] was employed to process Kyoto Encyclopedia of Genes and Genomes (KEGG) pathway enrichment analysis.

### 2.8 Active-subnetwork-oriented pathway enrichment analyses

The R package pathfindR (version 1.6.4) was applied to process active-subnetwork-oriented pathway enrichment analyses [32]. The gene names, log_2_ (Fold change) and P values of differentially expressed proteins (DEPs, P < 0.05) were imported into pathfindR. The parameters for pathfindR processing were: PIN, KEGG database; Gene set, gene ontology-biological process (GO-BP); and enrichment threshold = 0.05.

### 2.9 Immunoblotting

Immunoblotting (IB) was performed as we previously described [25], [26], [27], [28]. Briefly, the Blot Mini Gel Tank and Blot Module Set (Thermo) with precast polyacrylamide gels of NuPAGE 10% Bis−Tris protein gels (1.5 mm × 10 wells, Thermo) were used. Primary antibodies included rabbit anti-ISG15 mAb (Sinobiological, Beijing, China; 1:2000), rabbit anti-syntenin mAb (Abcam, Shanghai, China; 1:1000), rabbit anti-TSG101 mAb (Merck, Guangzhou, China; 1:2000), rabbit anti-CD9 pAb (Abcam; 1:2000), and rabbit anti-GRP78/BIP pAb (Proteintech, Wuhan, China; 1:2000). HRP-conjugated anti-rabbit IgG mAb (CST; 1:20000) was used as the secondary antibody.

### 2.10 Extracellular vesicle isolation and characterization

The 8E5 cells (CRL-8993, ATCC, Virginia, USA) and CCRF-CEM cells (CEM, GDC0219, CCTCC, Wuhan, China) were cultured in the complete RPMI 1640 medium (Thermo), containing 1% penicillin/streptomycin (Thermo), and 10% fetal bovine serum (FBS, VivaCell, Denzlingen, Germany) at 37 °C, 5% CO_2_. To isolate extracellular vesicles (EVs), cells were seeded in 75 cm^2^ - flasks at 1 × 10^6^ cells/mL culturing in RPMI without FBS for 24 h. Cell supernatant was then collected and sequentially centrifuged at 220g for 10 min, and 15000g for 30 min at room temperature to remove cells and debris, respectively. Next, supernatant was filtered by a 0.22 µm Filters (Sartorius, Guangzhou, China), followed by EV harvesting through Amicon Ultrafilters with 30 kDa molecular weight cut-off (Merck Millipore, Guangzhou, China).

As we previously described, the NanoSight NS300 analyzer (Malvern, Shanghai, China) was employed to determine the size distribution and concentration of EVs [24], [27]. Specifically, EVs were diluted with PBS to a concentration among 1–10 × 10^8^ particles/mL and the camera level was set to 12. EVs were also subjected to transmission electron microscopy (TEM) observation and protein marker purity examination as we previously described [24], [27].

### 2.11 Quantification of cell-associated HIV-1 RNA

We employed an HIV-1 Cell-Associated RNA (CA-HIV RNA) Detection Kit (SUPBIO, Guangzhou, China) to quantify HIV-1 RNA carried by EVs secreted by 8E5 cells (8E5 EVs). This kit targets the 3′UTR region of HIV-1 RNA that quantifies various spliced and unspliced HIV-1 RNA forms [33], [34], [35]. In each PCR analysis, a strong positive control, a weak positive control, and a water negative control provided by this kit were used. RNA was extracted from 3 × 10^9^ 8E5 EVs and analyzed by this qPCR kit according to the manufacturer’s instructions with a SLAN 96-P Real Time PCR System (Hongshitech, Shanghai, China).

### 2.12 Preparation and characterization of SC-macrophages (SCM)

The SC cell is a non-cancerous human monocytic cell line that can be differentiated into resting macrophages (SC-macrophages, SCM) [36]. To prepare SCM, 2 × 10^5^ SC cells (CRL-9855, ATCC, Virginia, USA) per well were plated in 6-well plates and cultured with complete RPMI supplemented with 10% FBS at 37 °C, 5% CO_2_. SC cells were exposed to 200 nM phorbol-12-myristate-13-acetate (PMA, Sigma-Aldrich) for 3 d to allow monocytic differentiation, followed by the removal of PMA and continued 5-day culture to obtain resting SCM.

Flow cytometry (FCM) was performed to measure the CD14 expression on SC cells and SCM. Cells were washed twice with PBS, and treated with Human Trustain FcX (BioLegend, Beijing, China) for 15 min to block the Fc receptors. Cells were then stained with FITC conjugated anti-human CD14 antibody or anti-mouse IgG isotype control antibody (Biolegend) for 20 min in dark. For phagocytosis analysis, cells were treated with 2 mL 0.5 µm FluoSpheres carboxylate-modified microspheres (505/515, 1.68 × 10^8^ particles/mL, Thermo). After PBS washes, cells were resuspended and filtered by 70 µm sieve meshes. A FACSCelesta™ Cell Analyzer (BD Biosciences, Shanghai, China) was used for all FCM analyses.

### 2.13 RNA extraction and qPCR

SCM cells were treated with 8E5 or CEM EVs for 24 h, followed by total RNA extraction using the Eastep® Super Total RNA Extraction Kit (Promega, Shanghai, China). RNA was reversely transcribed to cDNA with the Hifair® III 1st Strand cDNA Synthesis SuperMix for qPCR (YEASEN, Shanghai, China). The qPCR was performed with the SsoAdvanced Universal SYBR Green Supermix (Bio-Rad, Guangzhou, China) on a CFX96™ System (Bio-Rad). All gene-specific primers used in this study were identical to Nasr et al. [37].

### 2.14 Statistics

For DEPs determination, Student’s t test or Kolmogorov-Smirnov test (KS-test) were used based on the Jarque-Bera normal test (JB-test) with MATLAB software (version 2016b, The MathWorks, Beijing, China). MATLAB software was employed for principal component analysis (PCA) and hierarchical clustering (distance = Euclidean, linkage = ward). GraphPad Prism version 6.01 (GraphPad Software, La Jolla, CA, USA) was used to perform Student’s t test. The density plot, violin plot and volcano plot analyses were performed by the ggplot2 R package (version 3.3.6, https://ggplot2.tidyverse.org).

## 3 Results

### 3.1 Proteome of HIV-1-associated colorectal cancer

**Fig. 1:**
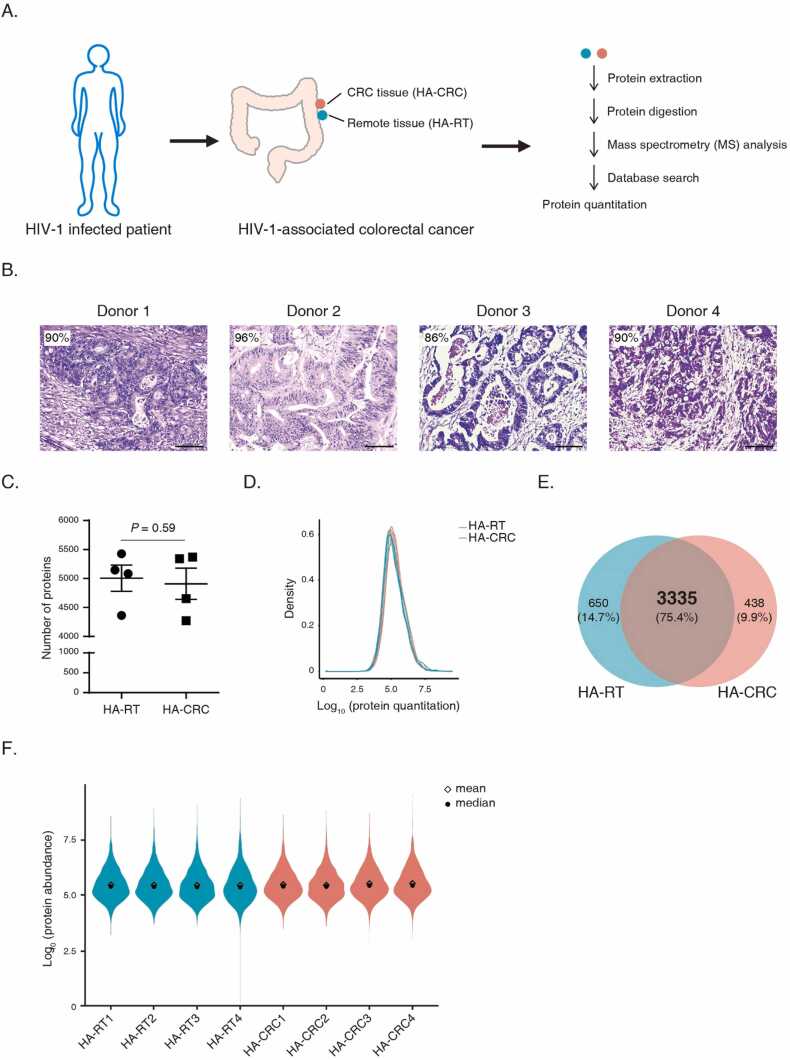
Summary of the mass spectrometry analysis on HIV-associated colorectal cancer and HIV-associated remote tissues. (A) Experimental design. (B) Image of HA-CRC tissue. Sections were stained with hematoxylin and eosin. Scale-bar = 200 µm. The cellularity is indicated in the left corner of each image. (C) Comparison of quantified protein numbers. Data are shown with mean± s.e.m., paired Student’s t test, n = 4. (D) Protein abundance distribution. The density plots of protein abundances are shown. (E) Venn diagram comparison of quantified proteins. (F) Abundance distribution of the commonly quantified proteins across all samples. The codes started with HA-RT and HA-CRC represent samples from remote tissue and HA-CRC primary tumor tissue, respectively.

In this study, we obtained HA-CRC and HA-RT tissue samples from four donors, followed by MS analysis (Fig. 1A). The HA-CRC samples were of high quality with confirmative CRC morphology for diagnosis. The cancer cellularity of the four donors was between 86% and 96% (Fig. 1B). We established a DDA proteome spectral library, consisting of 96,630 precursors, 66,453 peptides and 8615 protein groups. For each HA-CRC sample, approximately 4900 proteins were quantified. There was no significant difference in terms of the number of quantified proteins when comparing HA-CRC with HA-RT (Fig. 1C). As shown in density distribution plots, we observed normal distribution for protein quantifications in each sample (Fig. 1D). There were 3335 proteins that could be commonly quantified in all HA-RT and HA-CRC samples without missing values (Fig. 1E). The abundance distribution of these 3335 proteins was normal-like in each sample (Fig. 1F). Detailed information of the 3335 proteins can be found in Supplemental Table S1. The above data suggested that the quality of MS data was acceptable for subsequent analyses. All MS raw data can be downloaded from iProX database [38] (Accession number: IPX0005207000).

### 3.2 Clinical relevance of the proteome of HA-CRC and HA-RT

**Fig. 2:**
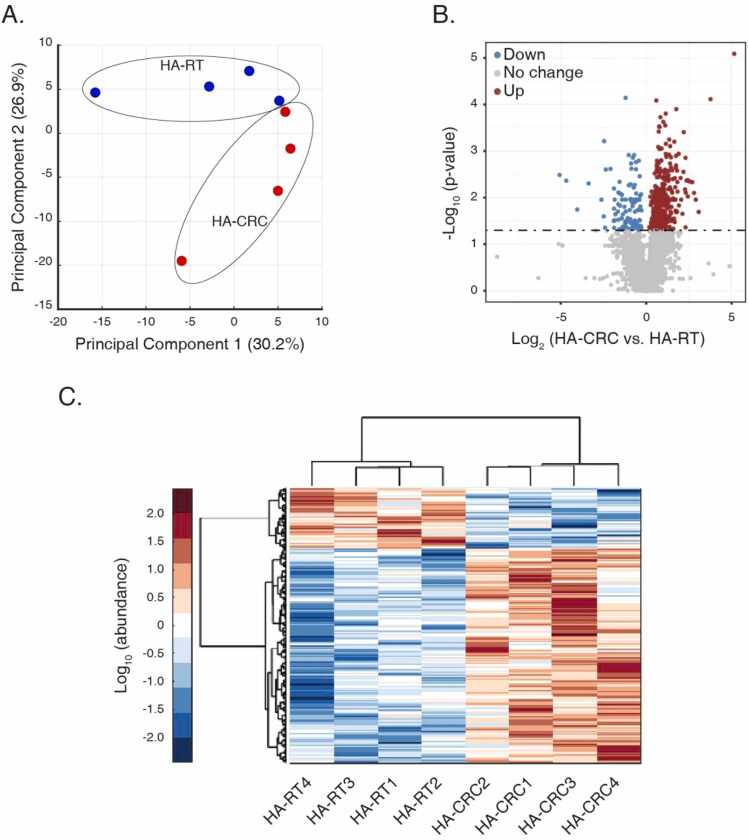
Pathological association of tissue proteomes. (A) Principal component analysis (PCA) with all quantified proteins. (B) Determination of DEPs comparing the abundance fold changes of HA-CRC vs. HA-RT. The p-values were obtained from Student t test or Kolmogorov-Smirnov test per normal tests. DEPs threshold was set as P = 0.05, and significantly up- and down- regulated DEPs in HA-CRC are shown in red and blue, respectively. (C) Hierarchical clustering with Euclidean Distance of DEPs, linkage = ward.

We used all the 3335 commonly quantified proteins to perform PCA analysis. HA-CRC and HA-RT tissue samples could be separated in a two-dimensional space formed by PC1 and PC2, with the variances of 30.2% and 26.9%, respectively (Fig. 2A). To be noted, PCA could not clearly separate the two groups, potentially due to the sample size limitation. Through the Student’s t test or KS-test, as compared with the paired HA-RT tissue, we identified 363 (10.9%) and 104 (3.1%) significantly up- and down- regulated DEPs in HA-CRC among the 3335 commonly quantified proteins (P < 0.05; Fig. 2B). Detailed information of these DEPs can be found in Supplemental Table S2. Using unsupervised clustering analysis, we noted that these 467 DEPs could well differentiate the HA-CRC and the HA-RT tissues (Fig. 2C). These analyses suggested that the proteome of HA-CRC and HA-RT tissues were of systematical in-group similarity and inter-group difference.

### 3.3 HA-CRC and non-HA-CRC share similar metabolic features per proteome

To discern the potential systems difference between HA-CRC and non-HA-CRC, we downloaded and re-analyzed the published data from CPTAC for comparison [21]. This CPTAC dataset contains isobaric tandem mass tag (TMT) labeling-based MS analysis results of 96 CRC tissues and their paired normal adjacent tissues (NAT). From this dataset, we extracted 4590 proteins that were quantified across all of the samples for further comparative analyses with all the 3335 quantified proteins in our current study.

**Fig. 3:**
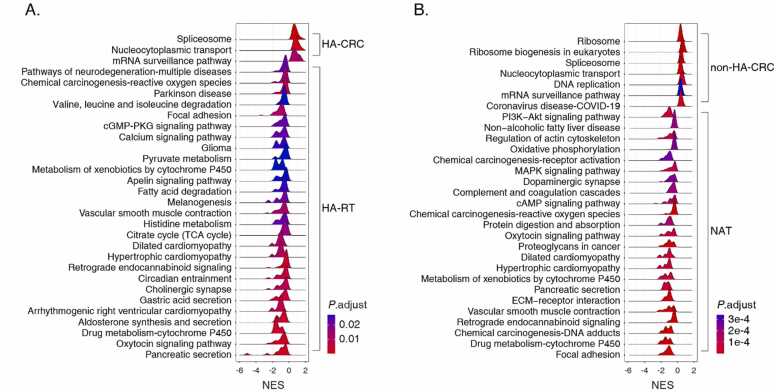
KEGG gene set enrichment analyses for all proteins quantified in both (A) HA-CRC and (B) non-HA-CRC by clusterProfiler. The proteome datasets of non-HA-CRC were downloaded from CPTAC [21].

By clusterProfiler analysis with the KEGG gene set, we found that spliceosome, nucleocytoplasmic transport and mRNA surveillance pathways were commonly over-represented in HA-CRC and non-HA-CRC (Fig. 3A and B). These key pathways are known to feature CRC in numerous studies that will be discussed in the following sections. Although multiple pathways were found to be enriched in HA-RT and NAT tissues, the fatty acid degradation, pyruvate metabolism, oxidative phosphorylation, protein digestion and absorption pathways suggested the relatively normal status of these non-cancerous colon tissues (Fig. 3A and Fig. 3B).

### 3.4 Antiviral response differentiated HA-CRC from non-HA-CRC

**Fig. 4:**
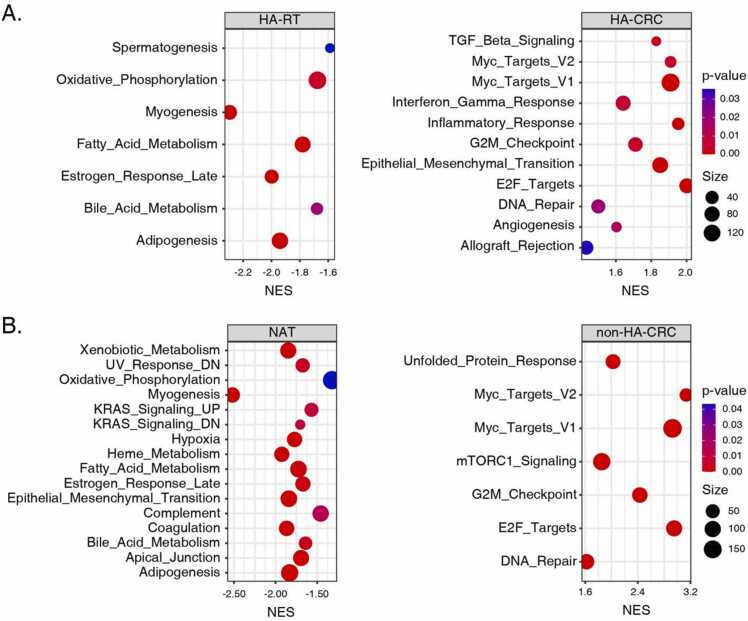
Hallmark gene set enrichment analyses for (A) HA-CRC and in (B) non-HA-CRC by GSEA. P < 0.05 was deemed significant enrichment, and the node size indicates the number of genes involved in a certain term.

The hallmark gene set is one of the Human Molecular Signatures Database (MSigDB) collections in GSEA [39]. While using the hallmark gene set as reference, typically physiological terms were significantly enriched in the HA-RT group, including oxidative-phosphorylation, myogenesis, metabolism for fatty and bile acids, and estrogen response (Fig. 4A). In contrast, significantly enriched terms for the NAT group (CPTAC dataset) combined both physiological ones similar to the HA-RT group and the cancer-associated ones, including K-ras signaling, epithelial-mesenchymal transition (EMT) and complement activation (Fig. 4B).

Interestingly, the enriched terms for HA-CRC indicated collective features of cancerous phenotypes and anti-viral response (Fig. 4A). The cancerous terms included transforming growth factor beta (TGF-β), Myc and E2F targets, G2M checkpoint, angiogenesis, and DNA repair (Fig. 4A). In addition, the interferon gamma (IFN-γ) and inflammation responses were among the top significantly enriched terms in HA-CRC (Fig. 4A). The detailed enrichment results can be found in Supplemental Table S3.

In comparison, the analysis on the non-HA-CRC (CPTAC dataset) featured commonly known cancerous hallmarks, but with no anti-viral response indications (Fig. 4B). To be noted, the unfolded protein response (UPR) was enriched as the top term, and the aberrant translation associated mTOR pathway was also over-represented (Fig. 4B). Similar to HA-CRC, the terms of G2M checkpoint, DNA repair, and Myc and E2F targets were commonly enriched in the non-HA-CRC.

### 3.5 Cancerous features in non-HA-CRC extracted from sub-network analysis on DEPs

**Fig. 5:**
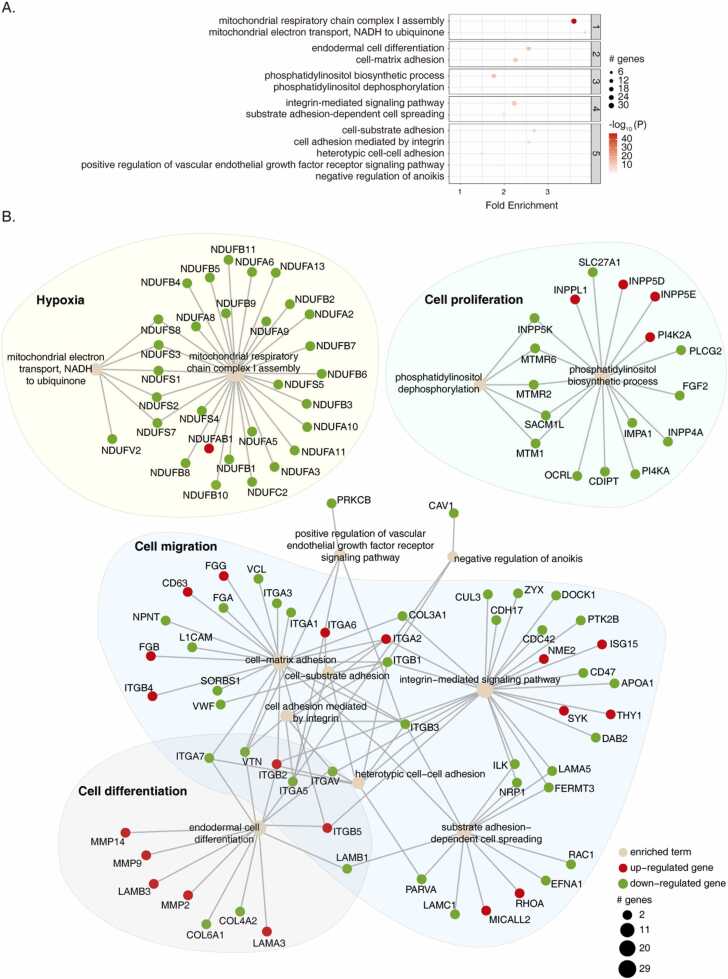
Active-subnetwork-oriented pathway enrichment of DEPs in non-HA-CRC. (A) Bubble chart of enrichment results of non-HA-CRC, grouped by clusters. The x axis denotes the fold enrichment value, while the y axis indicates the significantly enriched terms (enrichment threshold =0.05). The bubble size represents the number of DEPs in a certain term. The color scale corresponds to the -log_10_ (lowest-P) value. (B) Consolidated active subnetworks of non-HA-CRC. The up- and down- regulated DEPs were indicated in red and green, respectively. The Top 5 clusters per p-value ranking and their corresponding sub-networks are shown.

We next performed active-subnetwork-oriented pathway enrichment analysis on DEPs of the CPTAC dataset (Fig. 5). The purpose of this analysis was to serve as a background of the systems feature of non-HA-CRC for the subsequent comparison with HA-CRC. As compared with NAT tissues, 2095 and 1907 DEPs were found up- and down- regulated in the non-HA-CRC tissues. Following the pathfindR workflow, 1627 DEPs (40.7%) were found to have protein-protein interactions recorded in the KEGG Pathway database, which resulted in identifications of 46 clusters of enrichment terms and 69 active sub-networks (Supplemental Table S4). With respect to cluster rankings, we found that pathways associated with cancer hallmarks were among the top 5 clusters (Fig. 5A). Specifically, these pathways were regulating oxidative phosphorylation, differentiation, cell adhesion and proliferation (Fig. 5 A).

By consolidating the sub-networks, we could further extract the key DEPs and discriminate their collective roles in non-HA-CRC (Fig. 5B). This analysis suggested the top alteration of the mitochondria function, featured by the down-regulation of 28 proteins in mitochondrial respiratory chain complex I assembly that implicated the hypoxia status (Fig. 5B). Five proteins inducing the phosphatidylinositol dephosphorylation were significantly down-regulated suggesting the activation of PI3K pathway and promoted proliferation (Fig. 5B). Endodermal cell differentiation was significantly enriched based on 7 up-regulated DEPs including MMP14, MMP2 and ITGB5, and 7 down-regulated DEPs, such as COL4A4 and COL6A1 (Fig. 5B). Six significantly enriched terms focused on regulating cell-adhesion and integrin-mediated signaling pathway, based on 47 up- and down-regulated DEPs, including ITGA6 and RhoA (Fig. 5B).

### 3.6 Crosstalk of antiviral response and cancerous pathways in HA-CRC discerned

**Fig. 6:**
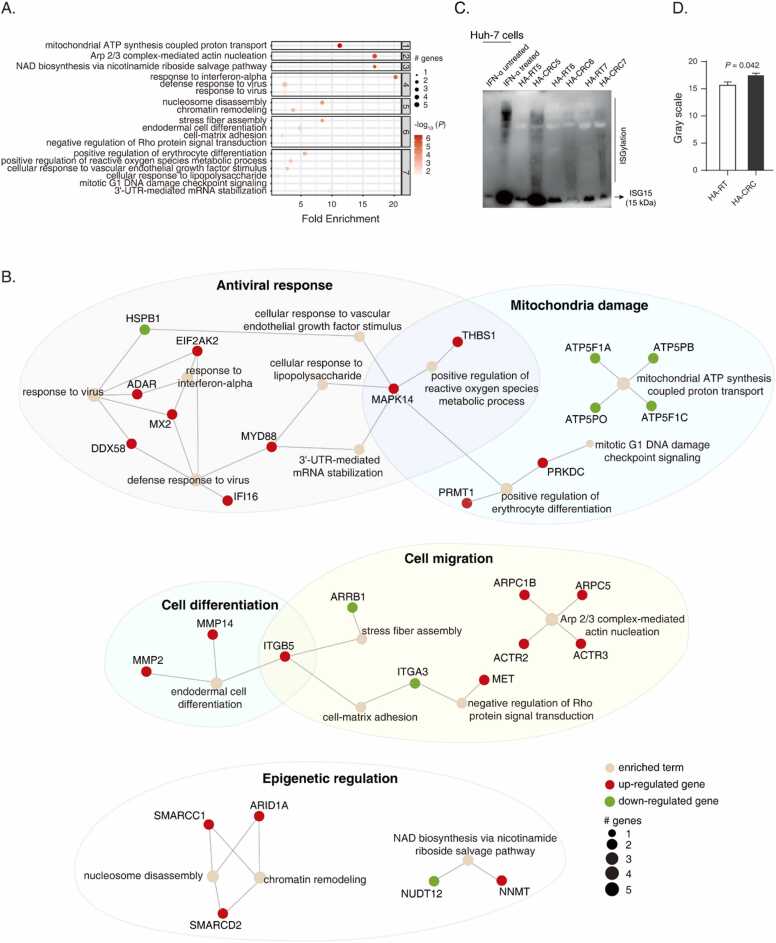
Active-subnetwork-oriented pathway enrichment of DEPs in HA-CRC and biological verification. Bubble chart of enrichment results of HA-CRC (A), and consolidated active subnetworks of HA-CRC (B) are shown. Please refer to the legend of Fig. 5 for other analytical settings and specifications. All significantly enriched terms and their corresponding sub-networks are shown. (C,D) IB analysis on ISGylated proteins comparing the HA-CRC with the HA-RT tissues (C), and the gray scale statistics (D). Huh-7 cells treated with and without 500 U/mL IFN-α were used as positive and negative controls, respectively.

Using similarly analytical procedure to Section 3.5, we ran pathfindR with 467 DEPs of HA-CRC, among which 185 DEPs had KEGG recorded protein-protein interactions. Seven clusters with 18 significantly enriched terms were found (Fig. 6). Comparing with the non-HA-CRC group, antiviral response and its associated inflammation were found to be significantly enriched (Fig. 6A), which involved in the up-regulation of EIF2AK2, ADAR, MX2, DDX58, MYD88 and IFI16, as well as the down-regulation of HSPB1 (Fig. 6B). The reactive oxygen species (ROS) pathway and mitochondria dysfunction were connected via MAPK14. Notably, four proteins of mitochondrial respiratory chain complex V were down-regulated (Fig. 6B). Similar to non-HA-CRC, the endodermal cell differentiation pathway was significantly enriched based on MMP2, MMP14 and ITGB5 (Fig. 6B). In addition, actin nucleation regulating pathways such as Arp2/3 was significantly activated per up-regulated ARPC5, ARPC1B, ACTR3 and ACTR2 (Fig. 6B). Finally, the epigenetic regulation tended to be over-represented in HA-CRC, as implicated the upregulation of SMARCC1, SMARCD2, ARID1A and NNMT (Fig. 6B).

To prove whether antiviral response was over-represented in HA-CRC, we further enrolled Donors 5–7 to perform biological verifications. It is known that protein ISGylation is a critical marker of the activation of IFN signaling pathway [40]. With reference to Rueschenbaum et al. [41], we used IFN-α (500 U/mL) to induce the protein ISGylation in a human liver cell line Huh-7 cells that serves as a positive control (Fig. 6C), in which Coomassie Brilliant Blue R-250 staining was performed to control the sample loading amount (Supplemental Fig. S1). Post IFN-α treatment, Huh-7 cells showed remarkable up-regulation of ISGylated proteins (Fig. 6C). We found that as compared with HA-RT tissues, the HA-CRC tissues showed significantly up-regulated protein ISGylation (Fig. 6C and 6D).

### 3.7 EVs from 8E5 cells are HIV-1 RNA positive and induce antiviral response in macrophages

All HA-CRC Donors of this study had achieved viral suppression, it is interesting to trace the sources of the antiviral response. In PLWH, the majority of HIV-1 DNA is defective proviral DNA [42], [43]. Defective HIV-1 DNA is not silenced, rather they are transcriptional and/or translational active [44]. In addition, EVs have been recognized as an important compartment that carries and transfers cell-derived biomolecules, including RNA and proteins[45]. As such, we posit that defective HIV-1 reservoir can release viral RNA through EVs to induce antiviral response in target cells. Furthermore, macrophages are known to be the primary immune cells that recognize viral RNAs followed by the activation of IFN pathways to defend virial infections [37], [46]. To address this hypothesis, we employed 8E5 cells to represent defective HIV-1 reservoir cells. 8E5 cells are known to be derived from an acute lymphoblastic leukemia cell line CEM cells. An 8E5 cell is integrated with a single-copy HIV-1 genome that is defective in the pol region.

**Fig. 7:**
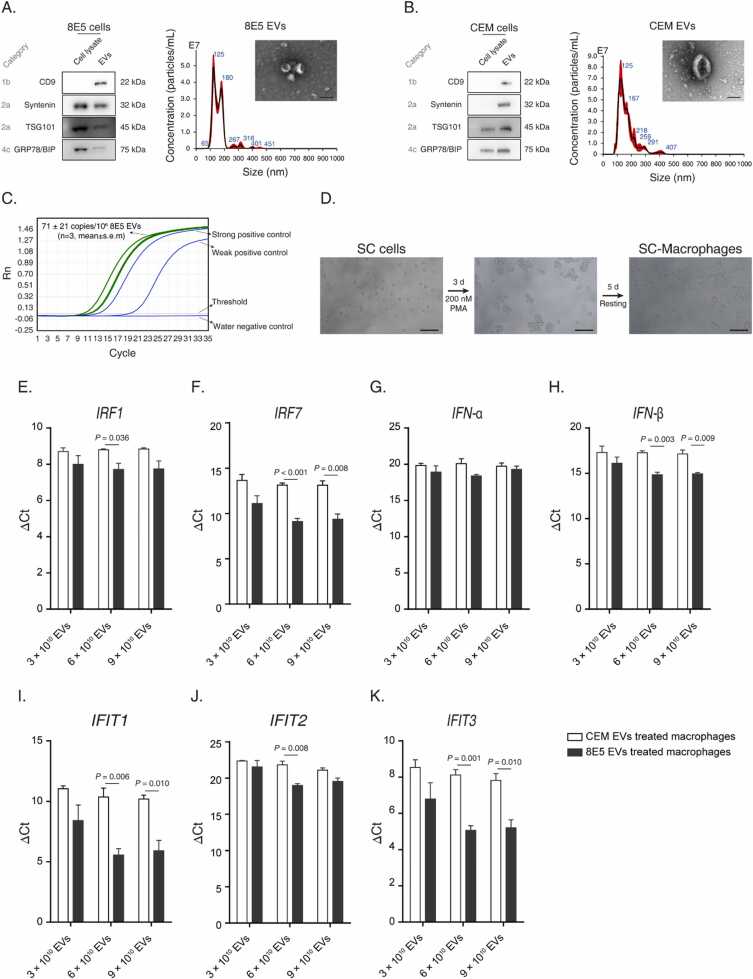
Defective HIV-1 reservoir cells elicit anti-viral response in macrophages via extracellular vesicles carrying cell-associated HIV-1 RNA. (A,B) Characterization of extracellular vesicles (EVs) isolated from 8E5 cells (A) and CEM cells (B). EVs were subjected to IB, NTA and TEM analyses. Scale bar = 100 nm. (C) qPCR analysis of CA-HIV RNA carried by 8E5 EVs. (D) Preparation of SC-macrophages. Scale bar = 100 µm. (E∼K) qPCR analysis on antiviral response - associated genes in EV-stimulated SCM. SCM were exposed to different amounts of EVs isolated from 8E5 or CEM cells. The expression of IRF1 (E), IRF7 (F), IFN-α (G), IFN-β (H), IFIT1 (I), IFIT2 (J) and IFIT3 (K) were measured by qPCR, in which ΔCt = Ct (target gene) - Ct (GAPDH). All results were acquired from three independent biological replications. Data are shown with mean± s.e.m. Statistical comparison was performed by the unpaired Student’s t test.

First, we isolated EVs from 8E5 and CEM cells, followed by the examination of EV purity according to the MISEV2018 recommendations published by International Society of Extracellular Vesicles [47]. Specifically, both 8E5 EVs (Fig. 7A) and CEM EVs (Fig. 7B) showed positive for EV markers of CD9, TSG101 and syntenin, while the EV co-isolating contaminant marker BIP (HSPA5) was largely removed. In addition, the diameter ranges of 8E5 and CEM EVs were 60–200 nm, and small cup-shaped particles could be observed via TEM (Fig. 7A and 7B). The particle protein ratio of 8E5 and CEM EVs were (3.0 ± 0.3) × 10^8^ and (3.8 ± 0.2) × 10^8^ particles/μg protein (mean± s.e.m, n = 3), respectively. The above results indicated that EVs had been isolated with high purity per MISEV2018.

Second, for CA-HIV RNA measurement, the strong and weak positive controls, and the water negative controls were all properly worked (Fig. 7 C), while the R (correlation coefficient) of the standard curve was> 0.999 (Supplemental Fig. S2). We found that 8E5 EVs carried 71 ± 21 copies CA-HIV RNA/1 × 10^6^ EVs (mean± s.e.m, n = 3, Fig. 7 C), while CEM EVs were CA-HIV RNA negative.

Next, we successfully prepared SCM according to the following results. Post PMA treatment for 3 d, suspended and round-shaped SC cells became attached and some cells formed protrusions (Fig. 7D). After removal of PMA and continued 5-day culture, typical spread morphology could be observed in most SCM (Fig. 7D). In addition, SCM showed significantly up-regulated CD14 expression (Supplemental Fig. S3A), and significantly more phagocytotic cells (Supplementary Fig. S3A) as compared with SC cells.

Finally, we treated SCM with different amounts of 8E5 or CEM EVs for 24 h, followed by the determination of the antiviral response associated gene alterations. We found that as compared with CEM EVs, 8E5 EVs (6 × 10^10^ and/or 9 × 10^10^ EVs) significantly increased the expression of IFN transcription factors IRF1 (Fig. 7E) and IRF7 (Fig. 7F) in SCM. Although no significant alteration was observed for the IFN-α expression comparing all experimental groups (Fig. 7G), IFN-β was found to be significantly up-regulated in the 6 × 10^10^ and the 9 × 10^10^ 8E5 EVs treated SCM groups (Fig. 7H). In addition, we found that the interferon-stimulated genes (ISGs) of IFIT1 (Fig. 7I), IFIT2 (Fig. 7J) and IFIT3 (Fig. 7K) were all significantly up-regulated in the 8E5 EVs treated SCM groups (6 × 10^10^ and/or 9 × 10^10^ EVs) as compared with the corresponding CEM EVs treated SCM groups.

## 4 Discussion

In the field of NADC, there are major obstacles for molecular mechanism exploration, especially due to the lack of in vitro and/or animal models to adequately reproduce the interactive functions of viral and host factors. Despite this, numerous groups have made promising advances to address this question via simplified models focusing on certain cell types and viral proteins/nucleic acids. While in this study, we tried to employ a holistic strategy with a high-quality proteomics analysis that discerned the crosstalk between antiviral response and cancerous pathways in HA-CRC, leading us to biologically reveal a critical role of EVs released from defective HIV-1 reservoir cells (8E5 cells) in activating IFN pathways in macrophages.

We performed restricted quality control in both sample acquisition and MS analysis. It is generally accepted that tissues with tumor cellularity of ≥50% is sufficient for clinical proteomics [48], [49]. In our previous report, we set a criteria of cancer cellularity of>85% that was proven to be important for subsequent proteome subtyping for liver cancer [23]. Accordingly, HA-CRC samples with the cellularity of>85% that were used in this study served as a stringent baseline control. Furthermore, we followed the recommendations of Human Proteome Project (HPP) MS Data Interpretation Guidelines 3.0, specifically employing peptide and protein FDR<0.01, and peptide length ≥9 aa for database search [50]. This tends to improve the evidence level for both protein identification and quantification that facilitates data sharing with the scientific community.

The subsequent whole proteome analysis allowed us to discern the general similarity and difference between HA-CRC and non-HA-CRC. First, the GSEA based on KEGG pathway implicated that the two types of CRC shared similarly over-represented pathways, including spliceosome, nucleocytoplasmic transport and mRNA surveillance that were known in CRC progression [51], [52]. The nucleocytoplasmic transport is a basic requirement for transcription and translation. We have previously shown that cancer cells are highly active in translation (Wang et al., 2013; Guo et al., 2015), which echoes the significantly enriched ribosome-associated terms in the non-HA-CRC group. Second, Hanahan has proposed fourteen hallmarks of cancer [53], and among them, three hallmarks are connected to the enriched terms in this study. These hallmarks included: activating invasion and metastasis (enriched terms in this study, TGF-β signaling, and EMT), sustained proliferation (c-Myc, E2F targets, G2M checkpoint, mTOR), genome instability and mutation (DNA repair), and tumor-promoting inflammation (inflammatory response, unfolded protein response).

The active sub-network analysis helped us to discern the systems mechanism and molecular-based network crosstalk implicated by the HA-CRC and non-HA-CRC proteome. Through reanalysis of the CPTAC dataset, we found massive downregulation of proteins that constitute mitochondria complex I, suggesting the hypoxia status of non-HA-CRC. This is comparable with Vasaikar et al. [21] showing that glycolysis pathway activation is important in the subtyping of advanced CRC. Phosphorylation of phosphatidylinositol by PI3K is a critical event for oncogenesis and cancer progression [54]. We noted that multiple proteins regulating dephosphorylation of phosphatidylinositol were down-regulated, suggesting their collective role for maintaining the PI3K pathway. In addition, numerous down-regulating proteins constituted the cell adhesion associated sub-networks, implicating the gain of migratory ability of CRC cells [55], [56]. Moreover, MMP2 [57], MMP14 [58] and ITGB5 [59] were differentiation and metastasis regulating proteins in CRC, while they were enriched in the sub-networks in both non-HA-CRC and HA-CRC.

However, we found very different molecular systems in HA-CRC as compared with non-HA-CRC. The antiviral response sub-network centered the IFN-mediated immune response in HA-CRC. This sub-network consisted of EIF2AK2, ADAR1, MX2, MYD88, DDX58 and IFI16, and all of these genes were interferon-stimulated genes (ISGs) that were critical to inhibit virus replication [60]. Such activated innate immunity sub-network tended to bridge the crosstalk of antiviral response and cancerous pathways. For example, MyD88 has been shown to promote to the proliferation, migration and invasion of cancer cells via MyD88/NF-κB/AP-1 signaling [61]. In addition, the mitochondria impairment in HA-CRC was also linked to the antiviral response associated subnetwork suggesting the hypoxia micro-environment (TME) conversion in HA-CRC [62].

We biologically verified the significant up-regulation of antiviral response in HA-CRC, as indicated by the protein ISGylation analysis. Indeed, chronic and weak activation of IFN pathways have beneficial effects to numerous cancer cell types [63]. On one hand, most cancer tissues are hypoxic, while necrotic cells can release DNA fragments into TME. These DNA can be recognized by cGMP-AMP synthase to induce IFN expression that contributes to the chronic IFN responses in cancer cells [63]. On the other hand, cancer cells can aberrantly express endogenous retroviral elements (ERE) that mimic pathogenic viral infection to maintain chronic IFN expression by immune cells in TME [64], [65].

Our findings implicated that defective HIV-1 reservoir derived EVs can transfer CA-HIV RNA to macrophages and induce the activation of IFN pathways. It should be noted that the all donors of this study had achieved HIV-1 RNA suppression prior to CRC surgery. Although plasma HIV-1 RNA is below the limits of detection, it is known that HIV-1 DNA intergraded in human genome can still actively transcribe CA-HIV RNA [66]. Through the mechanism revealed from our current study, HA-CRC does not need to solely use ERE, while the CA-HIV RNA contained in EVs released from HIV-1 reservoirs is of significance in maintaining the chronic activation of IFN pathways. Comparably, Fan et al. have found that IFN-induced protein ISGylation can intensify intestinal inflammation and colitis-associated CRC in mouse models [67]. Similar mechanisms in other infectious diseases are comparable to our study. For example, EVs released from HBV-infected hepatocytes have been found to contain viral nucleic acids that are associated with increased NKG2D ligand expression in macrophages by stimulating MyD88, TICAM-1, and MAVS-dependent pathways [68]. In addition, Dreux et al. have found the HCV-RNA containing EVs can induce innate immunity in plasmacytoid dendritic cells [69].

In conclusion, we have reported the first functional proteomic analysis on HA-CRC that systematically and biologically discerns the converged antiviral response and cancerous pathways. Defective HIV-1 reservoir cells as represented by 8E5 cells can induce the IFN pathway activation via horizonal transfer of CA-HIV RNA via EVs.

## CRediT authorship contribution statement

**T.W. and M.W.**： Conceived the idea and supervised the entire study. **Z.C. and K.Y.**：Designed and performed most of the experiments. **Z.C., J.Z., S.R.:** Contributed to the data analysis. **H.C.:** Performed pathological experiments and data analysis. **J.G. and Y.C.:** Contributed to study supervision, and data analysis. **T.W. Z.C., K.Y., J.Z., S.R., M.W.:** Contributed to manuscript preparation and paper writing. All authors reviewed and approved the manuscript.

## Declaration of Competing Interest

The authors declare that they have no known competing financial interests or personal relationships that could have appeared to influence the work reported in this paper.
