# Supplementary material for: Systems crosstalk between antiviral response and cancerous pathways via extracellular vesicles in HIV-1-associated colorectal cancer
Source: Comput Struct Biotechnol J. 2023 Jun 12;21:3369–82. doi: 10.1016/j.csbj.2023.06.010 (PMC10300105; doi:10.1016/j.csbj.2023.06.010)
Supplement: Supplementary file 1 — Supplementary material [file mmc1.docx]

Systems crosstalk between antiviral response and cancerous pathways via extracellular vesicles in HIV-1-associated colorectal cancer

Zimei Chen ^a, b, 1^, Ke Yang ^b, 1^, Jiayi Zhang ^a^, Shufan Ren ^a^, Hui Chen ^b^, Jiahui Guo ^a^, Yizhi Cui ^a^, Tong Wang ^a, b,^ *, Min Wang ^b,^ *

**Supplementary Figures**


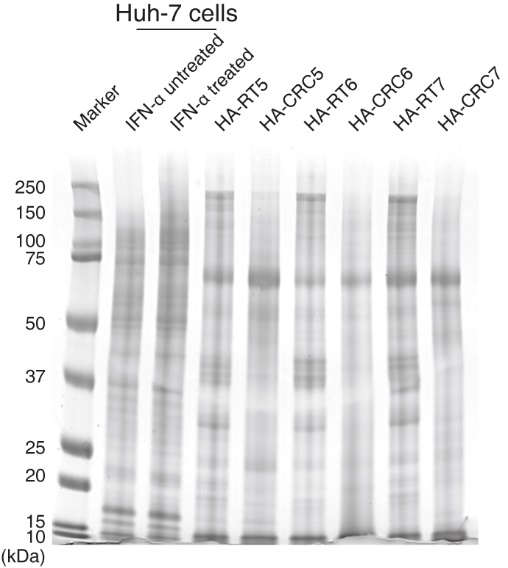


**Supplementary Figure S1.** Loading control. In each lane, 10 μg proteins was loaded and Coomassie Blue R-250 staining was performed.


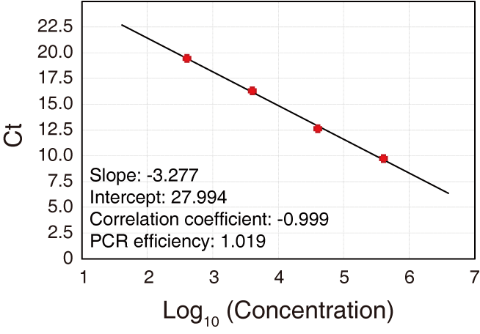


**Supplementary Figure S2.** The standard curve of the qPCR analysis of CA-HIV RNA.


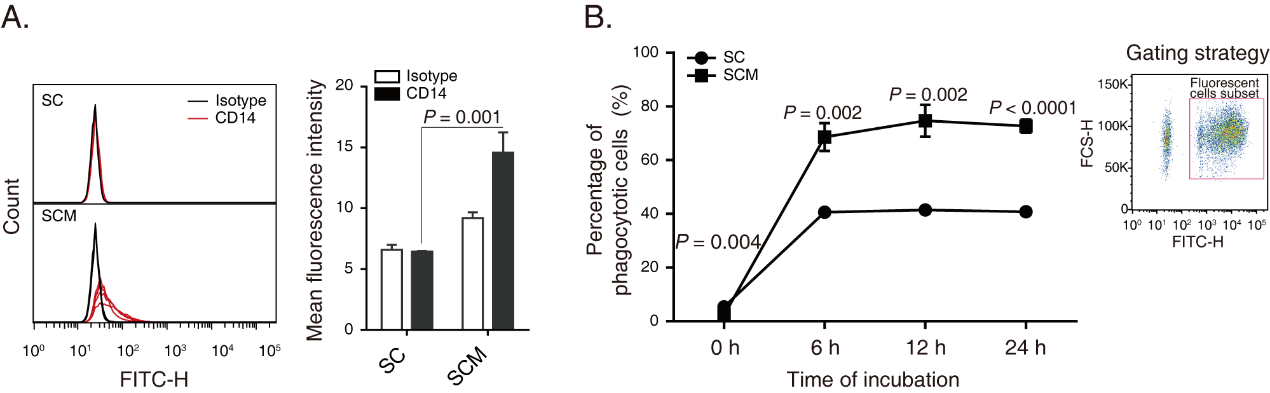


**Supplementary Figure S3.** Characterization SC-macrophages. (A) Flow cytometry (FCM) analysis on the expression of CD14. Mean fluorescence intensity of CD14 was statistically compared between SC cells and SC-macrophages (SCM). (B) Phagocytosis analysis. SC cells or SCM were treated with Fluorosphere and longitudinally analyzed with FCM. The gating strategy is shown to differentiate phagocytotic and non-phagocytotic cells. Results were obtained from five (A) and four (B) independent experiments. Data are shown with mean ± s.e.m. Statistical comparison was performed by the unpaired Student’s *t* test.
